# Biological Activity of Vegetal Extracts Containing Phenols on Plant Metabolism

**DOI:** 10.3390/molecules21020205

**Published:** 2016-02-08

**Authors:** Andrea Ertani, Diego Pizzeghello, Ornella Francioso, Anna Tinti, Serenella Nardi

**Affiliations:** 1 Dipartimento di Agronomia, Animali, Alimenti, Risorse Naturali e Ambiente (DAFNAE), Università di Padova, Viale dell’Università 16, 35020 Legnaro (Padova), Italy; andrea.ertani@unipd.it (A.E.); serenella.nardi@unipd.it (S.N.); 2 Dipartimento di Scienze Agrarie, Università di Bologna, Viale Fanin 44, 40127 Bologna, Italy; ornella.francioso@unibo.it; 3 Dipartimento di Scienze Biomediche e Neuromotorie, Università di Bologna, Via Belmeloro 8/2, 40126 Bologna, Italy; anna.tinti@unibo.it

**Keywords:** vegetal extract, biostimulant, maize, hormone, phenolic acid, phenylpropanoid

## Abstract

The influence of vegetal extracts derived from red grape, blueberry fruits and hawthorn leaves on *Zea mays* L. plant growth and the activity of phenylalanine ammonia-lyase (PAL), a key enzyme of the phenylpropanoid pathway, was investigated in laboratory experiments. The extracts were characterized using FT-IR and Raman spectroscopies in order to obtain a pattern of the main functional groups. In addition, phenols content was determined by HPLC, whereas the content of indoleacetic acid and isopentenyladenosine hormones was determined by ELISA test and the auxin and gibberellin-like activities by plant-bioassays. The treated maize revealed increased root and leaf biomass, chlorophyll and sugars content with respect to untreated plants. Hawthorn, red grape skin and blueberry at 1.0 mL/L induced high *p*-coumaric content values, whilst hawthorn also showed high amounts of gallic and *p*-hydroxybenzoic acids. PAL activity induced by hawthorn at 1.0 mL/L had the highest values (11.1-fold UNT) and was strongly and linearly related with the sum of leaf phenols. Our results suggest that these vegetal extracts contain more than one group of plant-promoting substances.

## 1. Introduction

European agricultural and food safety policies are promoting the safe use of agricultural inputs with low environmental impact in response to consumer demands for healthy food products. In the last few years plant growth biostimulants, being active at low dosages, have had rapidly increasing success on the agricultural inputs market as a “softer” agricultural practice to replace or complement mineral fertilizers [1,2,3]. They thus represent a relevant alternative solution for improving crop quality while reducing environmental pollution. 

Biostimulants are produced from a wide range of materials (humic substances, seaweeds, plants, living microbial cultures, protein hydrolysates and amino acids, and synthetic molecules) that may operate at different metabolic level favoring a better assimilation, translocation and use of nutrients [4]. However crop species and cultivar, development stage, environmental conditions (*i.e.*, temperature, relative air humidity) and dose, time and method of biostimulant application may influence the final effect [1,2]. The biosynthesis of phenolic compounds are among the possible directly involved metabolic pathways [5,6,7,8]. Most phenolic compounds have shown important properties such as their ability to act as antioxidants in protecting the body against reactive oxygen species or as regulators of plant growth rates [9] or soil processes, as well as decomposition and nutrient recycling [10], and have a by-product in their antiherbivore activity [11].

In general the biosynthetic pathways to phenylpropanoids and related phenolics are known and phenylalanine ammonia-lyase (PAL; EC 4.3.1.5) enzyme catalyzes the first committed step by converting phenylalanine to *trans*-cinnamic acid and tyrosine to *p*-coumaric acid. Nevertheless, the factors regulating and controlling the quality and quantity of phenols in plant tissues still remain controversial. Much of this controversy arises from the many factors regarding the interaction between genotype and environment. This has led to a wide variation of plant phenol production among and within species over time.

Protocatechuic, hydroxybenzoic, vanillic and *p*-coumaric acids have been recognized as potential allelopathic agents. They influence membrane perturbation, which is followed by a cascade of physiological effects that include improvement of plant-water relationships, stomatal function and rate of photosynthesis and respiration. These phenols also interact with several phytohormones and enzymes determining a different biosynthesis and flow of carbon into metabolites [12]. Cytokinins have positive effects on biosynthesis/accumulation of specific phenolic acids with therapeutic value during the *in vitro* propagation of *Merwilla plumbea* [13]. Moreover, protocatechuic, caffeic and chlorogenic acids have significant antioxidant, anti-free radical, immunostimulating and anticancer activities [14]. *p*-Hydroxybenzoic acid exhibits antimicrobial, antifungal, antisickling and estrogenic activities [15]. Vanillic acid exhibits antisickling and anthelmintic activities, while syringic acid, besides being an antioxidant, shows antibacterial and hepatoprotective activities [16]. In this work we explore the applications of vegetal extracts from hawthorn (*Crataegus monogyna* Jacq.) leaves, red grape (*Vitis vinifera* L.) skin material and blueberry (*Vaccinium vitis-idaea* L.) fruits on the growth of maize plants and their effects on sugar and phenolic metabolism. We hypothesize that the presence of phenols in vegetal extracts should stimulate natural processes to enhance nutrient efficiency, tolerance to abiotic stress, and crop quality.

## 2. Results

### 2.1. Chemical and Spectroscopic Characterization of Vegetal Extracts

The chemical characteristics of the vegetal extracts are listed in Table 1.

**Table 1:** Chemical characteristics, auxin and gibberellin-like (IAA-like and GA-like) activities, and content of indoleacetic acid (IAA) and isopentenyladenosine (IPA) hormones in hawthorn (HN), red grape skin (RGS) and blueberry (BB) extracts.

Variable	HN	RGS	BB
pH	2.9	2.9	2.9
Total sugars (g/L)	8.4 ^b^*	5.7 ^c^	12.4
Gallic acid (mg/L)	4.5 ^a^	2.63 ^b^	n.d.
Chlorogenic acid (mg/L)	20.1b	n.d.	46.0 ^a^
Vanillic acid (mg/L)	3.6 ^b^	8.84 ^a^	n.d.
Caffeic acid (mg/L)	21.8 ^a^	n.d.	4.85 ^b^
*p*-Coumaric acid (mg/L)	2.2	n.d.	n.d.
*p*-Hydroxybenzoic acid (mg/L)	150.8 ^b^	152.3 ^b^	303.5 ^a^
Total phenolic acids (mg/L)	1140 ^b^	970 ^c^	4830 ^a^
IAA-like ^†^	1.50·10^−2^	n.d.	n.d.
GA-like ^†^	1.09·10^−7^ ^b^	1.34·10^−6^ ^a^	n.d.
IAA (nMol)	14.6 ^a^	11.6 ^b^	14.9 ^a^
IPA (nMol)	4.1 ^a^	4.91 ^a^	2.9 ^b^

n.d., not detectable. ^†^ mg/L hormone corresponding to 1.0 mL/L of vegetal extracts. * In each row, mean values with different letters significantly differ for *P* ≤ 0.05 by Student-Newman-Keuls test.

**Figure 1:**
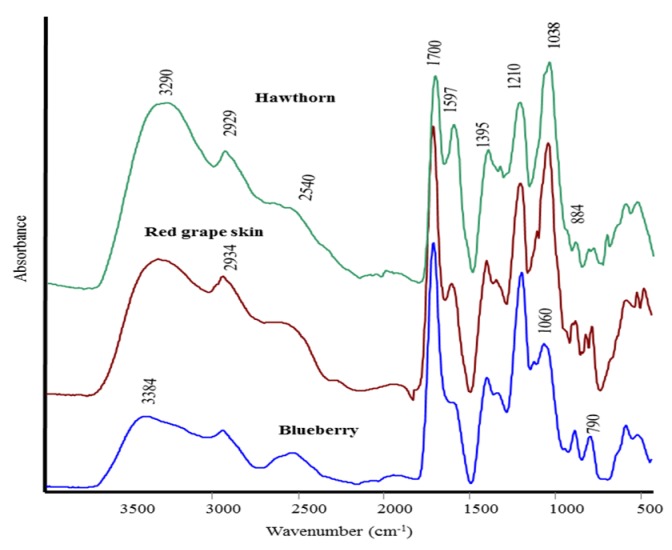
FT-IR spectra of lyophilized hawthorn leaves, red grape and blueberry fruits.

The pH value of extracts was the same, whereas their composition differed significantly. Blueberry had 2-fold and 5-fold higher total sugar and total phenolic acid contents than red grape skin (*P* ≤ 0.05), respectively, while hawthorn fell in an intermediate position.*p*-Hydrozybenzoic acid was dominant among the phenolic compounds, with a content in blueberry 2-fold that in red grape skin and hawthorn (*P* ≤ 0.05). A relatively high caffeic acid content was found in hawthorn and chlorogenic acid respect to blueberry. *p*‑Coumaric acid was found only in hawthorn. Concerning hormones, indoleacetic acid (IAA) was high in hawthorn and blueberry, whilst isopentenyladenosine (IPA) was elevated in red grape skin and hawthorn. Hawthorn also exhibited IAA-like activity, whereas both hawthorn and red grape skin possessed a somewhat gibberellin-like (GA-like) activity. Figure 1 shows the FT-IR spectra of hawthorn leaves (HN), red grape (RGS) and blueberry fruits (BB) while Table 2 reports the most significant Raman bands of the same samples. 

In the IR spectra the presence of OH groups was indicated by a broad band at around 3200 cm^−1^ and at 1040–1060 cm^−1^ (C-OH stretching /bending of primary or aromatic alcohols) [17,18]. The carboxylic acid carbonyl group shows two strong bands at 1700 cm^−1^ and 1210 cm^−1^ that may be assigned to asymmetric stretching of C=O and –C-OH stretching, respectively. The strong band at 1395 cm^−1^, together, partially, with the band at 1597 cm^−1^, can be attributed to ionized carboxylic acids (*i.e.*, COO groups). Finally, the weak shoulder at 1100 cm^−1^, the strong band at 1040–1060 cm^−1^ (C-O stretching; C-C stretching; C-CH bending) and the bands at 880 and 790 cm^−1^ (C-OH, C-CH and O-OH bending) are related to β anomeric configuration of carbohydrates. The appearance of absorptions around 1600 cm^−1^ (C=C ring stretching) and 900–800 cm^−1^ (C-H out-of-plane bending) may be due to the aromatic compounds vibrations [17]. The spectra of all samples are rather similar, even if slight differences in relative intensities can be observed. In particular, BB showed the most intense bands at 1700 and 1210 cm^−1^, typical of carbonyl group in acids. In HN the strong band at about 1040 cm^−1^ may be attributed to amorphous silica, a component of the leaf [19]. Amide I (1680–1650 cm^−1^ ) and amide II (1550–1520 cm^−1^) bands typical of proteins were not observed.

The Raman spectra of all samples showed a medium-weak band at about 1730 cm^−1^ typical of ester groups, present in chlorogenic acid (hydroxycinnamic acid esters). Moreover, RGS and BB displayed an intense peak at 1642 cm^−1^, indicating the existence of aliphatic C=C double bonds. In agreement with the FT-IR results no evidence of proteins was observed. Other bands observed at 1628, 1604 and 1577 cm^−1^ typical of aromatic C=C bonds, were more intense in HN according to the FT-IR spectrum; BB is second for intensity and RGS last. The presence of both aliphatic and aromatic C=C bonds is observed in polyphenols, as in the case of the caffeic and isoferulic acids [20]. In agreement with the relative intensity of aliphatic and aromatic double bonds, the vegetal extracts showed a different polyphenols composition. The medium bands appearing in the 1290–1266 cm^−1^ region were attributed to the ν(C–O), characteristic of phenolic compounds, as also detected by FT-IR spectrum. The very intense band at 1154 cm^−1^ (together with the 1180 cm^−1^ one in the case of HN) and the medium band at 3060 cm^−1^ are attributable to aromatic compounds, in particular to those para-di substituted The 1154 cm^−1^ band is also characteristic of polyenes, such as carotenoids and resveratrol [21]. The weak and medium bands appearing in the 1100–900 cm^−1^ region are attributed to δ(CH) of the aromatic moieties and ν(C-C) of the aliphatic parts. The bands observed at 900–600 cm^−1^ are attributable to skeletal vibrations, *i.e.*, vibrations involving δ(CCC) motions. Also in this region, the vegetal extracts displayed a medium intensity band at 795 cm^−1^ typical of para-di substituted benzene. Finally, the medium-weak band appearing at 370 cm^−1^ in the Raman spectra of RGS and BB (less evident in the case of HN) is attributed to δ(CCO) bending vibrations in polyphenols and also to δ(CC=C) bending of the vinylidene group [21]. 

**Table 2:** The main bands observed in the FT-Raman spectra of vegetal extracts: hawthorn (HN), red grape skin (RGS) and blueberry (BB) in the 3100–3000 and 1800–400 cm^−1^ region.

Attribution	HN	RGS	BB
*p*-substituted benzene	3060 m	3060 m	3060 m
aliphatic esters (νC=O)		1730 w	1728 m
fumaric acid, aliphatic ketones (νC=O)	1694 w	-	1711 m
ν(C=C) allyl derivatives	-	1642 vs	1639 vs
ν(C=C) aromatic	1628 vs	1627 vs	1628 vs
δNH_2_ + ν(C=C) aromatic	1604 vs	1604 sh	1604 s
ν (C=C) aromatic	1577 sh		
δCH_3_/CH_2_	1446 m	1448 m	1446 m
ν_s_ COO^−^ δCH_3_	1406 m, sh 1378 m	1385 m	1406 m 1387 m
νC-O (phenolic)	1290 m-s 1266 m, sh	1267 m	1290 m 1266 m
δCH (aromatic) (*p*-disubstituted benzene)/polyenes	1180 1154 m	1154 vs	1154 m
δCH (aromatic) + ν(C-C) aliphatic	1084 m	1089 w	1076 w
δCH (aromatic)	1002 m	1008 m-s	1002 m-s
δCH (aromatic) + ν(C-C) aliphatic	947 vw	926 w	939 m, l
skeletal vibrations	896 w 873 w	871 w	888 w 873 w
skeletal vibrations (*p*-disubstituted benzene)	795 m-w	795 m-w	795 m
skeletal vibrations	681 w	681 m-w	-
skeletal vibrations (benzene *p*-disubstituted)	617 w	618 w	617 w
*n*-alkanes			420 w
δ(CCO) polyphenols and/or resveratrol		370 m-w	370 m-w

s = strong, m = medium, w = weak, v = very, sh = shoulder, l = large.

### 2.2. Effect of Vegetal Extracts on Plant Growth

**Figure 2:**
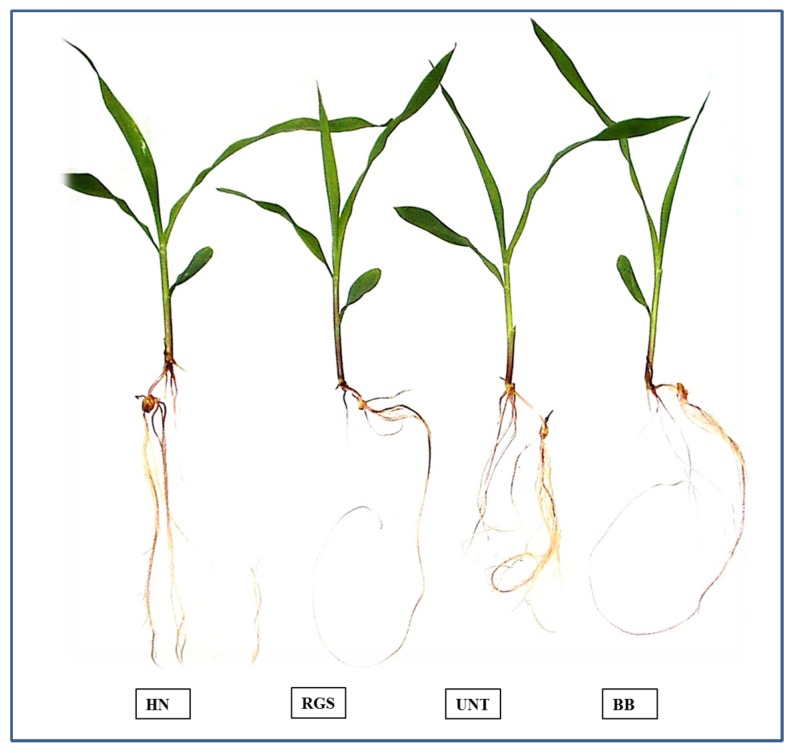
Fourteen days-old maize plants untreated (UNT) and treated with hawthorn leaves (HN), red grape skin (RGS) and blueberry fruits (BB).

Figure 2 shows the treated and untreated plants.

ANOVA analysis revealed that the dry weight, protein, glucose and fructose contents in maize plants were significantly affected by the treatment (*P* ≤ 0.001) and concentration (*P* ≤ 0.001) of vegetal extracts. 

**Table 3:** Dry weight, protein, glucose and fructose content ( ± SD) in roots of 14 days-old maize plants treated with hawthorn (HN), red grape skin (RGS) and blueberry (BB) extracts at two doses (0.1 and 1.0 mL/L).

Treatment	mL/L	Dry Weight	Protein	Glucose	Fructose
		g	mg/g f.w.	mg/g d.w.	
UNT	-	0.92 ± 0.01 ^Aaa^*	1.30 ± 0.01 ^Cd*c*^	3.25 ± 0.01 ^Ab*a*^	0.67 ± 0.02 ^Ce*b*^
HN	0.1	0.85 ± 0.01 ^b^	1.54 ± 0.04 ^b^	3.90 ± 0.01 ^a^	1.09 ± 0.02 ^c^
	1.0	0.91 ± 0.01 ^ab^	1.54 ± 0.07 ^b^	3.17 ± 0.01 ^b^	1.65 ± 0.01 ^a^
		0.88 ± 0.01 ^B^	1.54 ± 0.04 ^A^	3.54 ± 0.39 ^A^	1.37 ± 0.31 ^A^
RGS	0.1	0.89 ± 0.01 ^b^	1.65 ± 0.04 ^a^	2.74 ± 0.01 ^c^	1.23 ± 0.02 ^b^
	1.0	0.94 ± 0.01 ^a^	1.33 ± 0.03 ^d^	1.64 ± 0.01 ^d^	0.48 ± 0.01 ^e^
		0.91 ± 0.03 ^A^	1.49 ± 0.17 ^A^	2.19 ± 0.60 ^B^	0.85 ± 0.41 ^B^
BB	0.1	0.92 ± 0.01 ^a^	1.42 ± 0.01 ^c^	3.87 ± 0.01 ^a^	0.73 ± 0.02 ^de^
	1.0	0.88 ± 0.01 ^b^	1.31 ± 0.09 ^d^	2.96 ± 0.01 ^bc^	1.07 ± 0.01 ^c^
		0.90 ± 0.03 ^A^	1.37 ± 0.05 ^B^	3.42 ± 0.13 ^A^	0.90 ± 0.04 ^B^
	0.1	0.89 ± 0.03 *^b^*	1.54 ± 0.12 *^a^*	3.50 ± 0.53 *^a^*	1.02 ± 010 *^a^*
	1.0	0.91 ± 0.04 *^ab^*	1.39 ± 012 *^b^*	2.59 ± 076 *^b^*	1.07 ± 0.51 *^a^*

UNT, untreated = control. * In the same column, different letters indicated differences among treatments (upper case letters), among doses (italics) and treatment × dose (lower case) at *P* ≤ 0.05 by Student-Newman-Keuls test.

In roots (Table 3), vegetal extracts mostly increased the protein and fructose content, showing values up to 1.2- (*P* ≤ 0.05) and 2-fold UNT (*P* ≤ 0.05), respectively. Instead a minor effect was observed on the dry weight and glucose content. The biostimulant effect was related to type and dose of vegetal extract. For instance, red grape skin at 0.1 mL/L and hawthorn at 1.0 mL/L induced the highest amount of protein (1.3-fold UNT) and fructose (2.5-fold UNT), respectively (treatment × concentration interaction significant at *P* ≤ 0.001) (Table 3). In leaves (Table 4), vegetal extracts gave increments up to 1.3-fold UNT for both protein and glucose (*P* ≤ 0.05), and 1.6-fold UNT for fructose (*P* ≤ 0.05), whereas only a slight effect was found for dry weight. In general, the lowest dose of vegetal extract led to a better response in terms of increments up to 1.4- and 1.6-fold UNT, for glucose and fructose (*P* ≤ 0.05). Among treatments, red grape skin at 0.1 mL/L determined the highest amounts of glucose and fructose (approx. 2-fold UNT) (treatment × concentration interaction significant at *P* ≤ 0.001).

The chlorophyll a and b and phenolic acids contents were significantly affected by treatment (*P* ≤ 0.001) and concentration (*P* ≤ 0.001) of vegetal extracts (Table 5). As a consequence of treatment, the chlorophyll a content followed the order: blueberry = hawthorn > red grape skin > untreated (*P* ≤ 0.05), whilst for chlorophyll b it was: red grape skin > blueberry > hawthorn > untreated (*P* ≤ 0.05). A significant dose-effect relationship was observed. High values of chlorophyll a content were produced at the 0.1 mL/L dose whilst the optimum for chlorophyll b was at 1.0 mL/L. Phenolic acids such as *p*-coumaric and gallic acids were the most present in treated plants (Table 5). A considerable increase of these acids was observed at the 1.0 mL/L dose of vegetal extracts with respect to untreated and low dose (*P* ≤ 0.05). The 0.1 mL/L dose led to increases in vanillic, caffeic and *p*-hydroxybenzoic acids (*P* ≤ 0.05). A significant interaction was found between treatment × concentration (*P* ≤ 0.05) for phenols content. Hawthorn, red grape skin and blueberry at 1.0 mL/L induced high values of *p*-coumaric content. Hawthorn at 1.0 mL/L also showed high amounts of gallic and *p*-hydroxybenzoic acids whereas vanillic and caffeic acids at the 0.1 mL/L dose. 

The activity of the PAL enzyme in leaves of maize plants was significantly (*P* ≤ 0.001) increased by vegetal extracts treatments and doses (Table 5). The better activity was achieved at the following doses: 1.0 mL/L > 0.1 mL/L > UNT (*P* ≤ 0.05). In particular, the plants grown in the presence of hawthorn at 1.0 mL/L increased the PAL activity by 11.1-fold with respect to UNT. PAL activity was linearly related with the sum of leaf phenols, and the best relationship was evidenced by hawthorn (R^2^ = 0.92, *P* ≤ 0.05).

**Table 4:** Dry weight, protein, glucose and fructose content ( ± SD) in leaves of 14 days-old maize plants treated with hawthorn (HN), red grape skin (RGS) and blueberry (BB) extracts at two doses (0.1 and 1.0 mL/L).

Treatment	mL/L	Dry weight	Protein	Glucose	Fructose
		g	mg/g f.w.	mg/g d.w.	
UNT	-	0.76 ± 0.01 ^Bcb^*	1.83 ± 0.03 ^Bc*b*^	0.67 ± 0.01 ^Bc*b*^	0.64 ± 0.01 ^Bc*c*^
HN	0.1	0.86 ± 0.01 ^a^	2.23 ± 0.09 ^ab^	0.91 ± 0.01 ^b^	0.98 ± 0.01 ^b^
	1.0	0.83 ± 0.01 ^b^	2.03 ± 0.15 ^b^	0.78 ± 0.01 ^c^	1.06 ± 0.01 ^b^
		0.85 ± 0.02 ^B^	2.13 ± 041 ^A^	0.85 ± 0.07 ^A^	1.02 ± 0.04 ^A^
RGS	0.1	0.88 ± 0.01 ^a^	2.41 ± 0.02 ^a^	1.41 ± 0.01 ^a^	1.45 ± 0.01 ^a^
	1.0	0.86 ± 0.01 ^a^	2.41 ± 0.02 ^a^	0.39 ± 0.01 ^e^	0.40 ± 0.01 ^d^
		0.87 ± 0.01 ^A^	2.41 ± 0.02 ^A^	0.90 ± 0.56 ^A^	0.92 ± 0.55 ^A^
BB	0.1	0.86 ± 0.01 ^a^	2.22 ± 0.12 ^ab^	0.55 ± 0.01 ^d^	0.61 ± 0.01 ^c^
	1.0	0.85 ± 0.01 ^a^	2.25 ± 0.06 ^ab^	0.52 ± 0.01 ^d^	0.66 ± 0.01 ^c^
		0.86 ± 0.03 ^A^	2.23 ± 0.09 ^A^	0.54 ± 003 ^C^	0.63 ± 0.05 ^B^
	0.1	0.87 ± 0.01 *^a^*	2.29 ± 0.11 *^a^*	0.96 ± 0.32 *^a^*	1.00 ± 0.23 *^a^*
	1.0	0.85 ± 0.01 *^ab^*	2.23 ± 0.35 *^a^*	0.56 ± 0.18 *^b^*	0.71 ± 0.30 *^b^*

UNT, untreated = control. * In the same column, different letters indicated differences among treatments (upper case letters), among doses (italics) and treatment × dose (lower case) at *P* ≤ 0.05 by Student-Newman-Keuls test.

**Table 5:** Chlorophylls, phenol acids content and phenylalanine ammonia-lyase activity (PAL) ( ± SD) in leaves of 14 days-old maize plants treated with hawthorn (HN), red grape skin (RGS) and blueberry (BB) extracts at two doses (0.1 and 1.0 mL/L).

Treatment		Chlorophylls mg/g f.w.		Phenolic Acids mg/g f.w.					PAL
	mL/L	a	b	Gallic	Vanillic	Caffeic	*p*-Coumaric	*p*-Hydroxybenzoic	nmol Cinnamic Acid /mg protein/min
UNT	-	2.17 ± 0.01 ^Bcc^*	4.23 ± 0.01 ^Bc*c*^	2.23 ± 0.13 ^Cd*c*^	n.d.	0.60 ± 0.10 ^De*c*^	6.20 ± 1.06 ^Ce*c*^	2.50 ± 0.50 ^Cc*c*^	8.4 ± 2.0 ^Dg*c*^
HN	0.1	2.63 ± 0.01 ^a^	4.23 ± 0.01 ^c^	12.21 ± 1.00 ^c^	42.27 ± 1.63 ^a^	12.43 ± 1.00 ^a^	34.63 ± 0.80 ^d^	6.87 ± 0.23 ^a^	54.3 ± 7.3 ^d^
	1.0	2.48 ± 0.01 ^b^	5.32 ± 0.02 ^b^	23.85 ± 2.33 ^a^	20.43 ± 0.85 ^c^	2.50 ± 0.17 ^d^	168.03 ± 2.40 ^a^	7.17 ± 0.40 ^a^	93.8 ± 6.2 ^a^
		2.56 ± 0.08 ^A^	4.78 ± 0.55 ^B^	18.03 ± 6.60 ^A^	31.35 ± 12.01 ^A^	7.47 ± 5.48 ^A^	101.33 ± 73.08 ^A^	7.02 ± 0.34 ^A^	74.1 ± 22.4 ^A^
RGS	0.1	2.58 ± 0.01 ^ab^	4.62 ± 0.04 ^c^	13.54 ± 0.97 ^c^	n.d.	6.68 ± 0.49 ^b^	15.37 ± 0.97 ^e^	5.90 ± 0.10 ^b^	17.9 ± 5.3 ^f^
	1.0	2.35 ± 0.04 ^bc^	6.07 ± 0.03 ^a^	22.70 ± 0.95 ^b^	n.d.	6.86 ± 0.14 ^b^	148.67 ± 7.77 ^b^	2.50 ± 0.30 ^c^	78.2 ± 3.2 ^c^
		2.47 ± 0.04 ^AB^	5.34 ± 0.01 ^A^	18.12 ± 4.97 ^A^	n.d.	6.77 ± 0.31 ^B^	82.02 ± 70.30 ^B^	4.20 ± 1.79 ^B^	48.1 ± 7.0 ^C^
BB	0.1	2.71 ± 0.02 ^a^	5.58 ± 0.01 ^b^	10.50 ± 0.55 ^c^	23.10 ± 1.30 ^b^	5.10 ± 0.40 ^c^	44.50 ± 3.12 ^d^	3.40 ± 0.66 ^c^	35.2 ± 2.2 ^e^
	1.0	2.42 ± 0.02 ^b^	4.94 ± 0.32 ^c^	18.03 ± 1.21 ^a^	26.40 ± 2.01 ^b^	3.00 ± 0.56 ^d^	170.03 ± 10.40 ^a^	2.30 ± 0.22 ^d^	90.0 ± 3.5 ^b^
		2.56 ± 0.02 ^A^	5.26 ± 0.34 ^A^	14.25 ± 0.76 ^B^	24.75 ± 3.40 ^B^	4.05 ± 1.40 ^C^	107.25 ± 8.32 ^A^	2.70 ± 0.36 ^C^	62.5 ± 2.4 ^B^
	0.1	2.69 ± 0.03 *^a^*	4.45 ± 0.24 *^b^*	12.08 ± 1.42 *^b^*	21.79 ± 2.66 *^a^*	8.07 ± 2.65 *^a^*	31.50 ± 5.21 *^b^*	5.39 ± 1.60 *^a^*	35.8 ± 7.3 *^b^*
	1.0	2.47 ± 0.13 *^b^*	5.12 ± 0.81 *^a^*	21.52 ± 1.90 *^a^*	15.61 ± 3.65 *^b^*	4.12 ± 1.11 *^b^*	162.23 ± 10.50 *^a^*	3.90 ± 0.50 *^b^*	87.3 ± 7.9 *^a^*

UNT, untreated = control. * In the same column, different letters indicated differences among treatments (upper case letters), among doses (italics) and treatment × dose (lower case) at *P* ≤ 0.05 by Student-Newman-Keuls test.

## 3. Discussion

The treatment of maize plants with vegetal extracts from hawthorn leaves, red grape skin material and blueberry fruits positively affected their growth and investigated metabolic pathways. The observed effects indicate the presence of more than one group of plant-promoting substances [22] and a different composition of phenolic compounds as also supported by FT-IR and Raman spectra. IAA and IPA hormones were found in all three vegetal extracts at different amounts. Moreover, IAA-like activity was shown in hawthorn, whilst there was a somewhat GA-like activity in hawthorn and red grape skin. 

After hawthorn, red grape skin and blueberry application, a sharp increase in the maize plant dry weight was found in both roots and leaves. This is a typical effect of substances with biostimulant activity that have the capacity to modify plant metabolic processes in order to improve the potential growth benefits [23,24,25,26,27]. 

From a metabolic viewpoint, natural biostimulants (*i.e.*, humic substances) modulate carbon and nitrogen metabolism increasing the enzymes involved in glycolysis, the Krebs cycle and nitrate assimilation [23,27]. Carbohydrates such as glucose and fructose are considered the basis of plant metabolism, providing the energy required for various metabolic pathways as well carbon skeletons for nitrogen metabolism. In this study, after the application of hawthorn, red grape skin and blueberry, the glucose and fructose content increased sharply in both roots and leaves. These data are consistent with previous reports showing that humic substances induced an increase in the carbohydrate metabolism [28,29], which was also observed in pepper plants treated with red grape skin [26].

Hawthorn, red grape skin and blueberry vegetal extracts application increased the content of protein and chlorophylls. The high proteins content is probably a result of an increment in N uptake. The enhancement of N assimilation was already been seen in other biostimulants containing phenolic acids [5]. Alternatively, this effect might also be due to the presence of IPA. In fact, it has been shown that humic substances containing IPA induced the production of N assimilates and promoted photosynthesis through an increase in the chlorophyll content and stimulation of ATP sulfurylase and *O*-acetylserine sulfhydrylase activities [30]. Indeed, cytokinins have a positive effect on photosynthesis through expression of the sulfur responsive genes [31,32]. However, treatment with humic substances has been shown to increase the S content in maize roots [33] and to induce an early stimulation of sulfate uptake in *Brassica napus* [34].

Other metabolic pathways involving secondary metabolites appear to be a consequence of the treatments [35]. Total phenolic acids were strongly enhanced in leaves after the vegetal extract application. In particular, hawthorn, red grape skin and blueberry stimulated phenylpropanoid metabolism, as confirmed by the increased PAL enzyme activity and the accumulation of some phenols in the leaves of maize plants. Single phenolic compounds showed changes in relation to the vegetal extract and dose. Gallic and *p*-coumaric acids sharply increased in the leaves after treatment with the 1 mL/L dose. Vanillic, caffeic and *p*-hydroxybenzoic acids were also stimulated in treated plants and were very low in untreated ones. An increase in phenols in plant tissue may enhance plant resistance to stress conditions [36]. Furthermore, they can be a source of important antioxidants for human health [37]: for example, caffeic and gallic acids inhibit carcinogenesis [14,38].

## 4. Materials and Methods 

### 4.1. Chemical and Spectroscopic Characterization of Vegetal Extracts

Three vegetal extracts manufactured by ILSA S.p.A. (Arzignano, Vicenza, Italy) were used: hawthorn (*Crataegus monogina* Jacq.) leaves were produced by fully controlled enzymatic hydrolysis, and the red grape skin material from common grapevine (*Vitis vinifera* L.) and blueberry fruits (*Vaccinium vitis-idaea* L.) were obtained by cool extraction [39]. The samples were lyophilized before spectroscopic characterization.

For hawthorn, red grape skin and blueberry the pH was determined in water (3:50 *w*/*v*) [40]. Total phenols and sugars were determined according to [41,5], respectively. In particular, the extracted phenols were filtered at 0.45 µm and directly analyzed by using a HPLC 2700 (Thermo Finnigan, San Jose, CA, USA) coupled with a 1806 UV/Vis (Thermo Finnigan, San Jose, CA, USA) detector. The stationary phase was constituted by the column (Supelcosil TM-LC 18) and precolumn (Pelliguard TM-LC 18) of Supelco (Sigma-Aldrich, Milan, Italy). The mobile phase (1L) was constituted by n-butanol (Sigma-Aldrich, Milan, Italy) (18 mL) and acetic acid (Sigma-Aldrich) 50% (1.5 mL). Phenolic compounds were separated at room temperature (loop 20 µL) and with a flux of 1.2 mL/min. The analyzer was a UV detector at 275 nm. Each run lasted 30 min. Phenolic acids were calculated using the calibration curve of gallic, ferulic, vanillic, protocatetic, caffeic, p-coumaric, p-hydroxybenzoic, syringic and chlorogenic acids purchased by Sigma-Aldrich (see Supplementary Materials, Figures S1–S5). The calibration curve for quantitative analysis was performed on the basis of the relationship between peak areas vs standard concentrations at four concentration (*n* = 4). The calibration curve showed a linear fitting with values of the R squared (R^2^) = 0.99. For reducing sugars determination, a sample of material was dried for 48 h at 80 °C, ground in liquid nitrogen and then 100 mg were extracted with 2.5 mL 0.1 N H_2_SO_4_. Samples were incubated in a heating block for 40 min at 60 °C and then centrifuged at 6000 g for 10 min at 4 °C. After filtration (0.2 μm, Membra-Fil® Whatman Brand, Whatman, Milan, Italy), the supernatants were analyzed by HPLC coupled to the refractive index detector (RI) (Perkin Elmer 410, Perkin Elmer, Norwalk, CT, USA). The soluble sugars were separated through an Aminex 87 C column (300 × 7.8 mm, BioRad, Segrate, Milan, Italy) using H_2_O as eluent at a flow rate of 0.6 mL/min.

Infrared spectral acquisition was performed on solid samples, previously lyophilized, using a Nicolet 5700 FT-IR equipped with a diamond attenuated total reflectance (ATR) accessory and a DTGS (Nicolet, Madison, WI, USA) detector. The total number of scans averaged for each spectrum was 100 with a resolution of 4 cm^−1^. The background spectrum was acquired in air. Spectra analysis was performed with Grams/386 spectral software (Galactic Industries Corp., Salem, NH, USA).

Raman spectra of the lyophilized samples were recorded in solid state with a Multiram FT-Raman spectrometer (Bruker, Optics, Ettlingen, Germany) equipped with a cooled Ge-diode detector. The spectral resolution was 4 cm^−1^ and there were 6000 scans for each spectrum (integration time about 4 h). The excitation source was a Nd^3+^-YAG laser (1064 nm, about 35 mW laser power on the sample) in the backscattering (180°) configuration. The low laser power was due to the brown color of the samples.

### 4.2. Hormone Content and Activity

The indole-3-acetic acid (IAA) and isopentenyladenosine (IPA) hormones content in the vegetal extracts was quantified by using enzyme linked immuno-sorbent assays (ELISA; Sigma, St. Louis, MO, USA) as previously described [30].

The IAA-like activity was estimated by measuring the reduction of watercress (*Lepidium sativum* L.) roots after the treatment with the IAA and vegetal extracts, while the gibberellin-like (GA-like) activity was determined by the increase in the epicotyls length of lettuce (*Lactuca sativa* L.) after GA and vegetal extracts application [42,43]. In detail, watercress and lettuce seeds were surface-sterilized by immersion in 8% hydrogen peroxide for 15 min. After rinsing five times with sterile distilled water, 10 seeds were placed on a sterile filter paper in a sterile Petri dish. For watercress, the filter paper was wetted with 1.2 mL of a 1 mM CaSO_4_ solution (control), or 1.2 mL of 20, 10, 1, and 0.1 mg/L IAA solution (Sigma, Milan, Italy) for the calibration curve, or 1.2 mL of a serial dilution of vegetal extracts. For lettuce, the experimental design was the same as for watercress except that the sterile filter paper was wetted with 1.4 mL instead of 1.2 mL, and the calibration curve was a progression of 100, 10, 1 and 0.1 mg/L GA solution (Sigma). The seeds were placed in a germination room in the dark at 25 °C. After 48 h for watercress and 72 h for lettuce, the seedlings were removed and the root or epicotyl lengths measured with a TESA-CAL IP67 electronic caliper (TESA, Renens, Switzerland) and Data Direct software, version 1 (ArtWare, Asti, Italy). The values obtained were the means of 20 samples and five replications, with the standard errors always 5% of the mean.

### 4.3. Plant Material and Growth Conditions

Seeds of *Zea mays* L. (var. DK C6286, DeKalb, Monsanto, St. Luis, USA) were soaked in distilled water overnight and then surface-sterilized in 5% (*v*/*v*) sodium hypochlorite for 10 min, while shaking. Seeds were left to germinate for 60 h in the dark, at 25 °C, on a filter paper wetted with 1 mM CaSO_4_ [44]. Germinated seedlings were transplanted into 3 L pots containing an aerated complete culture solution, at a density of 24 plants per pot. The nutrient solution was renewed every 48 h and had the following composition (µM): KH_2_PO_4_ (40), Ca(NO_3_)_2_ (200), KNO_3_ (200), MgSO_4_ (200), FeNaEDTA (10), H_3_BO_3_ (4.6), CuCl_2_ (0.036), MnCl_2_ (0.9), ZnCl_2_ (0.09), NaMoO_4_ (0.01). Plants were cultivated for 14 days inside a climatic chamber with a 14 h light/10 h dark cycle, air temperature of 27 °C/21 °C, relative humidity of 70/85%, and photon flux density of 280 mol/m^2^/s. Twelve days after transplanting, plants were treated without vegetal extracts (untreated, UNT) or with 0.1 mL/L or 1.0 mL/L of biostimulant for 48 h. Plants were randomly harvested from three pots per treatment, carefully washed and dried with blotting paper. A sub-sample of plant material was immediately frozen with liquid nitrogen and kept at –80 °C for physiological analyses. For fresh weight measurement, thirty plants per treatment were used (ten per pot). Plants were divided into roots and leaves, and weighed separately.

### 4.4. Protein Extraction

Soluble proteins were extracted from frozen leaf and root tissues (500 mg) ground in liquid nitrogen, vortexed with 5 mL extraction buffer (100 mM Tris HCl pH 7.5, 1 mM Na_2_EDTA, 5 mM DTT), and centrifuged for 15 min at 14,000 *g*. The supernatants were mixed with 10% (*w*/*v*) trichloroacetic acid and centrifuged. The pellets obtained were re-suspended in 0.1 N NaOH. The protein concentration was analyzed at λ = 595 nm [45] using a UV/VIS spectrophotometer (Lambda 1, Perkin-Elmer, Norwalk, CT, USA) and expressed in mg protein/g fresh weight.

### 4.5. Determination of Soluble Phenols and Sugars

The amount of phenolic and sugar compounds was determined on fresh tissue and dry tissue, respectively, in accordance with the literature [5,41,46] and following the methods reported in the paragraph 4.1. 

### 4.6. Determination of Chlorophyll Content

To determine the chlorophyll content, 300 mg of fresh leaf tissue from five representative plants per pot were ground in liquid nitrogen and extracted with 15 mL ethanol (96% *v*/*v*). The samples were kept in the dark for 2 days at 4 °C, and the extracts were filtered and then analyzed spectrophotometrically (UV/VIS Lambda 1; PerkinElmer, Norwalk, CT, USA) at λ = 665 nm for chlorophyll a and 649 nm for chlorophyll b. Chlorophyll concentration was calculated using the Wellburn and Lichtenthaler formula [47] and expressed in mg of pigment per g of leaf fresh weight.

### 4.7. Phenylalanine Ammonia-lyase Assay

Phenylalanine ammonia-lyase (PAL; EC 4.3.1.5) was extracted by homogenizing 1 g leaf tissue in 5 mL ice-cold 100 mM potassium-phosphate buffer (pH 8.0) containing 1.4 mM 2-mercaptoethanol and 0.10 g polyvinylpyrrolidone. After centrifuging at 4 °C for 15 min at 15,000 *g*, the supernatant was chromatographed on Sephadex G-25 (GE Healthcare UK, Buckinghamshire, UK) equilibrated with the same buffer. The eluate was the extracted enzyme used for the assay. A mixture of 0.4 mL of 100 mM Tris–HCl buffer (pH 8.8), 0.2 mL of 40 mM phenylalanine, and 0.2 mL of enzyme extract was incubated for 30 min at 37 °C and stopped with 0.2 mL 25% TCA [48]. Phenylalanine was added to the control after incubation and addition of the acid. After centrifuging at 4 °C for 15 min at 10,000 *g*, the absorbance of the supernatant was measured at 280 nm relative to the control. PAL activity is expressed as nmol cinnamic acid/mg protein/min. The enzyme activity of all the enzyme extracts was calculated relative to the protein concentration measured according to Bradford’s method [45].

### 4.8. Statistical Analyses

All examined variables were tested for normality and homoscedasticity (by Shapiro-Wilk’s and Levene's tests, respectively) and transformed when necessary to satisfy assumptions required by parametric statistics. Data were the means of three independent replicates. Analysis of variance (ANOVA) was performed using the SPSS software (SPSS, Chicago, IL, USA) and was followed by pairwise post-hoc analyses (Student-Newman-Keuls test) to determine which means differed significantly at *P* ≤ 5% [49].

## 5. Conclusions

Our study indicates that the presence of IAA and IPA hormones, GA-like activity and phenolic acids in vegetal extracts derived from red grape, blueberry fruits and hawthorn leaves is accompanied by high biostimulant activity. This is in accordance with previous finding [5,30,35], in which the biological activity of other plant growth biostimulants was studied. The IAA and auxin-like activity may be responsible for the stimulation of phenylpropanoid metabolism which occur through an auxin-mediated signal transduction. Alternatively, the effect on phenylpropanoid metabolism could be ascribed to other signaling molecules such as phenols. Indeed, phenolic acids may show hormone-like activity [39] and stimulate the phenylpropanoid pathway similarly to humic substances, which exert an auxin-mediated signal transduction [38]. Overall, phenolic acids may act independently via classical plant hormones, although a synergistic effect cannot be excluded.

## Supplementary Materials

Supplementary materials can be accessed at www.mdpi.com/1420-3049/21/2/205/s1.

## References

[1] Colla G., Nardi S., Cardarelli M., Ertani A., Lucini L., Canaguier R., Rouphael Y. (2015). Protein hydrolysates as biostimulants in horticulture. Sci. Hortic..

[2] Calvo P., Nelson L., Kloepper J.W. (2014). Agricultural uses of plant biostimulants. Plant Soil.

[3] Sharma H.S.S., Fleming C., Selby C., Rao J.R., Martin T. (2014). Plant biostimulants: A review on the processing of macroalgae and use of extracts for crop management to reduce abiotic and biotic stresses. J. Appl. Phycol..

[4] Du Jardin P. (2015). Plant biostimulants: Definition, concept, main categories and regulation. Sci. Hort..

[5] Ertani A., Schiavon M., Altissimo A., Franceschi C., Nardi S. (2011). Phenol-containing organic substances stimulate phenylpropanoid metabolism in *Zea mays*. J. Plant Nutr. Soil Sci..

[6] Szopa A., Ekiert H. (2014). Production of biologically active phenolic acids in *Aronia melanocarpa* (Michx.) Elliott *in vitro* cultures cultivated on different variants of the Murashige and Skoog medium. Plant Growth Regul..

[7] Aremu A.O., Plačková L., Gruz J., Bíba O., Novák O., Stirk W.A., Doležal K., van Staden J. (2015). Seaweed-derived biostimulant (Kelpak®) influences endogenous cytokinins and bioactive compounds in hydroponically grown *Eucomis autumnalis*. J. Plant Growth Regul..

[8] Aremu A.O., Stirk W.A., Kulkarni M.G., Tarkowská D., Turečková V., Gruz J., Šubrtová M., Pěnčík A., Novák O., Doležal K. (2015). Evidence of phytohormones and phenolic acids variability in garden-waste-derived vermicompost leachate, a well-known plant growth stimulant. Plant. Growth. Regul..

[9] Rice E.L. (1984). Allelopathy.

[10] Northup R.R., Yu Z., Dahlgren R.A., Vogt K.A. (1995). Polyphenol control of nitrogen release from pine litter. Nature.

[11] Inderjit (1996). Plant phenolics in allelopathy. Bot. Rev..

[12] Einhellig F.A., Macı`as F.A., Galindo J.C.G., Molinillo J.M.G., Cutler H.G. (2004). Mode of allelochemical action of phenolic compounds. Allelopathy Chemistry and Mode of Action of Allelochemicals.

[13] Aremu A.O., Gruz J., Šubrtová M., Szüčová L., Doležal K., Bairu M.W., Finnie J.F., Van Staden J. (2013). Antioxidant and phenolic acid profiles of tissue cultured and acclimatized Merwilla plumbea plantlets in relation to the applied cytokinins. J. Plant Physiol..

[14] Olthof M.R., Hollman P.C., Katan M.B. (2001). Chlorogenic acid and caffeic acid are absorbed in humans. J. Nutr..

[15] Chong K.P., Rossall S., Atong M. (2009). *In vitro* antimicrobial activity and fungitoxicity of syringic acid, caffeic acid and 4-hydroxybenzoic acid against *Ganoderma boninense*. J. Agr. Sci..

[16] Itoh A., Isoda K., Kondoh M., Kawase M., Watari A., Kobayashi M., Tamesada M., Yagi K. (2010). Hepatoprotective effect of syringic acid and vanillic acid on CCl_4_-induced liver injury. Biol. Pharm. Bull..

[17] Shulz H., Baranska M. (2007). Identification and quantification of valuable plant substances. Vib. Spectrosc..

[18] Socrates G. (1994). Infrared Characteristic Group Frequencies. Tables and Charts.

[19] Ribeiro da Luz B. (2006). Attenuated total reflectance spectroscopy of plant leaves: A tool for ecological and botanical studies. New Phytol..

[20] Sanchez-Cortes S., Garcia-Ramos J.V. (2000). Adsorption and chemical modification of phenols on a silver surface. J. Colloid Interf. Sci..

[21] Billes F., Ziegler I.M., Mikosch H., Tyihak E. (2007). Vibrational spectroscopy of resveratrol. Spectrochim. Acta A.

[22] Pizzeghello D., Cocco S., Francioso O., Ferrari E., Cardinali A., Nardi S., Agnelli A., Corti G. (2015). Snow vole (*Chionomys nivalis* Martins) affects the redistribution of soil organic matter and hormone-like activity in the alpine ecosystem: Ecological implications. Ecol. Evol..

[23] Nardi S., Carletti P., Pizzeghello D., Muscolo A., Senesi N., Xing B., Huang P.M. (2009). Biological activities of humic substances. Biophysico-Chemical Processes Involving Natural Nonliving Organic Matter in Environmental Systems. PART I. Fundamentals and impact of mineral-organic-biota interactions on the formation, transformation, turnover, and storage of natural nonliving organic matter (NOM).

[24] Ertani A., Pizzeghello D., Altissimo A., Nardi S. (2013). Use of meat hydrolyzate derived from tanning residues as plant biostimulant for hydroponically grown maize. J. Plant Nutr. Soil Sci..

[25] Ertani A., Pizzeghello D., Baglieri A., Cadili V., Tambone F., Gennari M., Nardi S. (2013). Humic-like substances from agro-industrial residues affect growth and nitrogen assimilation in maize (*Zea mays* L.) plantlets. J. Geochem. Explor..

[26] Ertani A., Pizzeghello D., Francioso O., Sambo P., Sanchez-Cortes S., Nardi S. (2014). *Capsicum chinensis* L. growth and nutraceutical properties are enhanced by biostimulants in a long-term period: Chemical and metabolomic approaches. Front. Plant Sci..

[27] Canellas L.P., Olivares F.L. (2014). Physiological responses to humic substances as plant growth promoter. Chem. Biol. Technol. Agricult..

[28] Nardi S., Pizzeghello D., Remiero F., Rascio N. (2000). Chemical and biochemical properties of humic substances isolated from forest soils and plant growth. Soil Sci. Soc. Am. J..

[29] Muscolo A., Panuccio M.R., Sidari M., Nardi S. (2005). The effects of humic substances on Pinus callus are reversed by 2,4-dichlorophenoxyacetic acid. J. Chem. Ecol..

[30] Pizzeghello D., Francioso O., Ertani A., Muscolo A., Nardi S. (2013). Isopentenyladenosine and cytokinin-like activity of different humic substances. J. Geochem. Explor..

[31] Maruyama-Nakashita A., Nakamura Y., Yamaya T., Takahashi H. (2004). A novel regulatory pathway of sulphate uptake in *Arabidopsis* roots: implication of CRE1/WOL/AHK4-mediated cytokinin-dependent regulation. Plant J..

[32] Hirai M.Y., Fujiwara T., Awazuhara M., Kimura Y., Noji M., Saito K. (2003). Global expression profiling of sulfur-starved *Arabidopsis* by DNA macroarray reveals the role of *O*-acetyl-l-serine as a general regulator of gene expression in response to sulfur nutrition. Plant J..

[33] Eyheraguibel B., Silvestre J., Morard P. (2008). Effects of humic substances derived from organic waste enhancement on the growth and mineral nutrition of maize. Bioresour. Technol..

[34] Jannin L., Arkoun M., Ourry A., Laîné P., Goux D., Garnica M., Fuentes M., Francisco S.S., Baigorri R., Cruz F. (2012). Microarray analysis of humic acid effects on *Brassica napus* growth: Involvement of N, C and S metabolisms. Plant Soil.

[35] Schiavon M., Pizzeghello D., Muscolo A., Vaccaro S., Francioso O., Nardi S. (2010). High molecular size humic substances enhance phenylpropanoid metabolism in maize (*Zea mays* L.). J. Chem. Ecol..

[36] Di Marco G., Gismondi A., Canuti L., Scimeca M., Volpe A., Canini A. (2014). Tetracycline accumulates in Iberis sempervirens L. through apoplastic transport inducing oxidative stress and growth inhibition. Plant Biol..

[37] Impei S., Gismondi A., Canuti L., Canini A. (2015). Document Metabolic and biological profile of autochthonous *Vitis vinifera L*. ecotypes. Food Funct..

[38] Raina K., Rajamanickam S., Deep G., Singh M., Agarwal R., Agarwal C. (2008). Chemopreventive effects of oral gallic acid feeding on tumor growth and progression in TRAMP mice. Mol. Cancer Ther..

[39] Machado S. (2007). Allelopathic potential of various plant species on downy brome: Implications for weed control in wheat production. Agron. J..

[40] Trinchera A., Natalini M., Sequi P. (2003). Regolamento CE n. 2003/2003 03.001 del Parlamento Europeo e del Consiglio del 13 Ottobre 2003 Relativo ai Concimi.

[41] Arnaldos T.L., Ferrer M.A., Garcia A.A.C., Muñoz R. (2001). Changes in peroxidase activity and isoperoxidase pattern during strawberry (*Fragaria × ananassa*) callus development. J. Plant Physiol..

[42] Audus L.J. (1972). Plant Growth Substances. Chemistry and Physiology.

[43] Pizzeghello D., Nicolini G., Nardi S. (2002). Hormone-like activities of humic substances in different forest ecosystems. New Phytol..

[44] Nardi S., Muscolo A., Vaccaro S., Baiano S., Spaccini R., Piccolo A. (2007). Relationships between molecular characteristics of soil humic fractions and glycolytic pathway and krebs cycle in maize seedlings. Soil Biol. Biochem..

[45] Bradford M.M. (1976). A rapid and sensitive method for the quantification of microgram quantities of protein utilizing the principle of protein-dye binding. Anal. Biochem..

[46] Pizzeghello D., Zanella A., Carletti P., Nardi S. (2006). Chemical and biological characterization of dissolved organic matter from silver fir and beech forest soils. Chemosphere.

[47] Wellburn A.R., Lichtenthaler H., Nijhorff M.E., Junk W. (1984). Formulae and program determine carotenoids and chlorophyll a and b of leaf extracts inferent solvents. Advances in Photosynthesis Research.

[48] Mori T., Sakurai M., Sakuta M. (2001). Effects of conditioned medium on activities of PAL, CHS, DAHP synthase (DS-Co and DS-Mn) and anthocyanin production in suspension cultures of *Fragaria ananassa*. Plant Sci..

[49] Sokal R.R., Rohlf F.J. (1969). Biometry.

