# Supplementary material for: Biological Activity of Vegetal Extracts Containing Phenols on Plant Metabolism
Source: Molecules. 2016 Feb 8;21(2):205. doi: 10.3390/molecules21020205 (PMC6273273; doi:10.3390/molecules21020205)
Supplement: Supplementary file 1 [file molecules-21-00205-s001.pdf]

Supplementary Material for: Biological activity on plant metabolism of vegetal extracts containing phenols

Andrea Ertani, Diego Pizzeghello [1,*], Ornella Francioso [2], Anna Tinti [3] and Serenella Nardi [1]

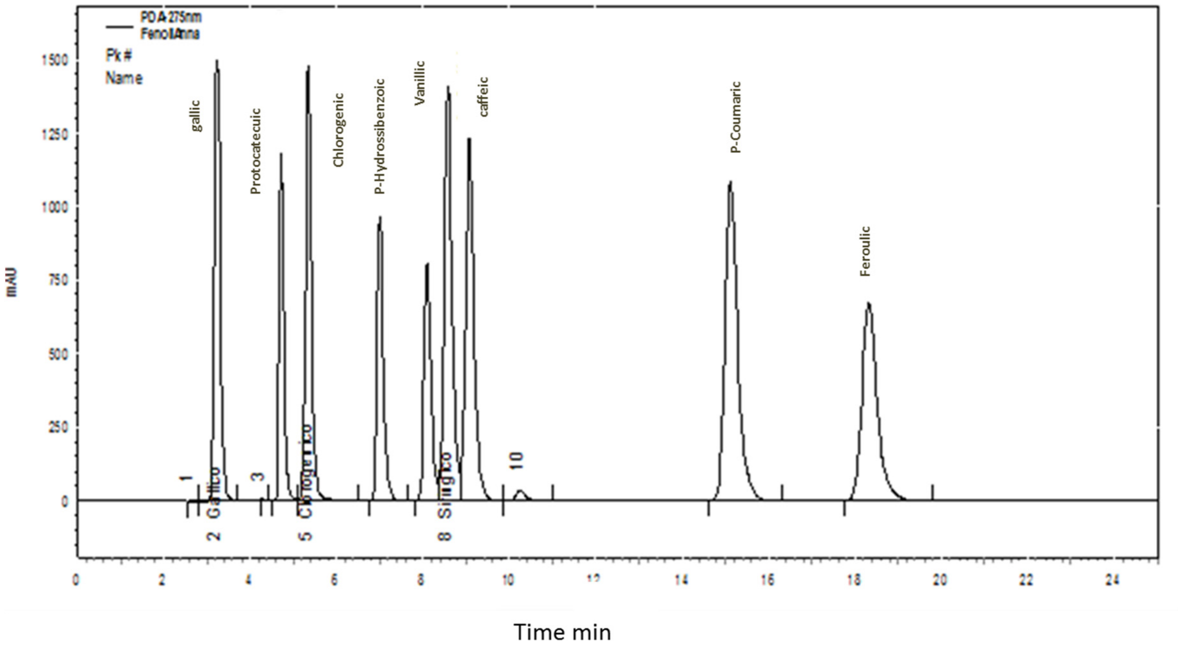

Figure S1. HPLC chromatogram with the separation of the standards of phenolic acids.

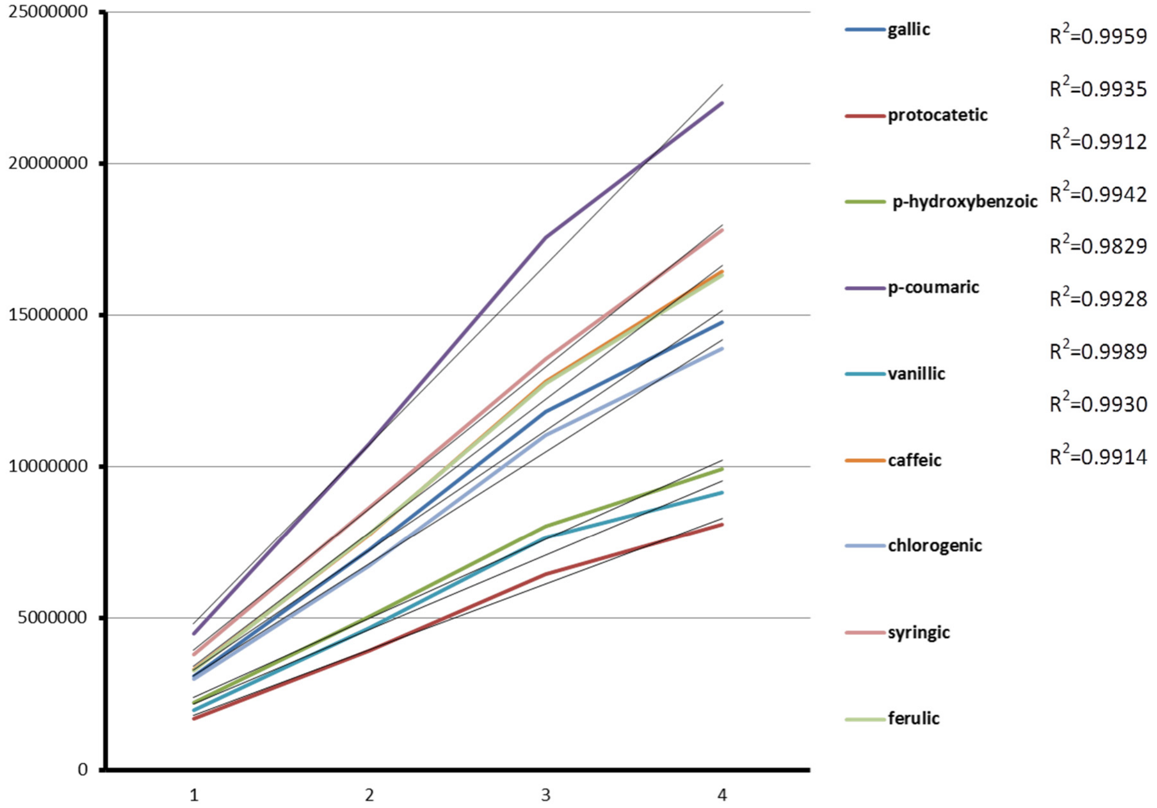

Figure S2. Calibration curves of the standards of phenolic acids

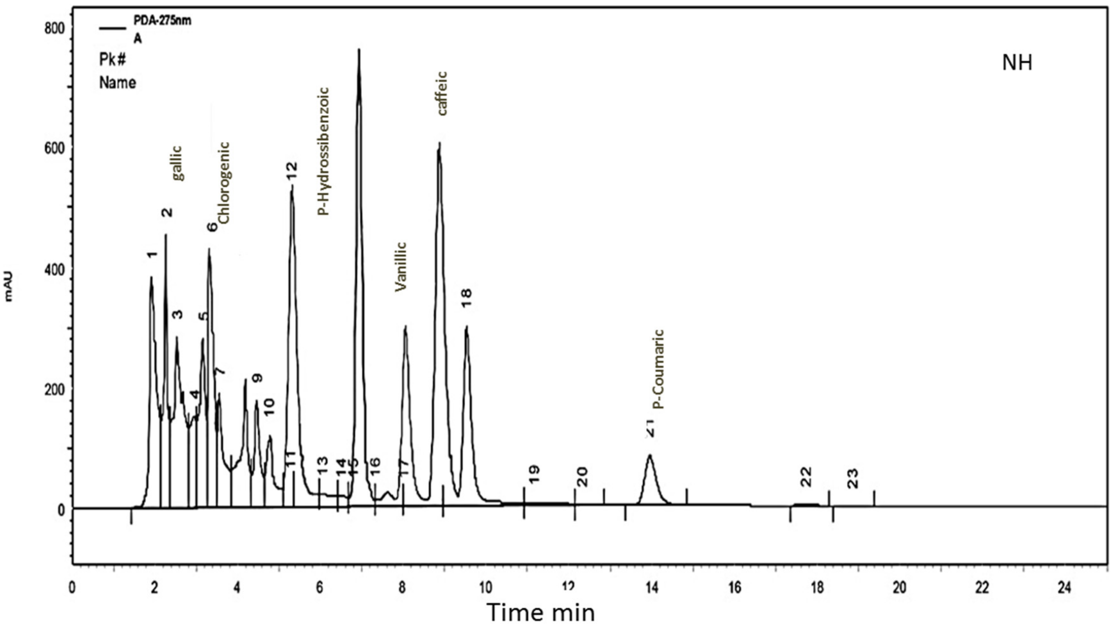

Figure S3. HPLC chromatogram of phenolic acids in Hawthorn leaves

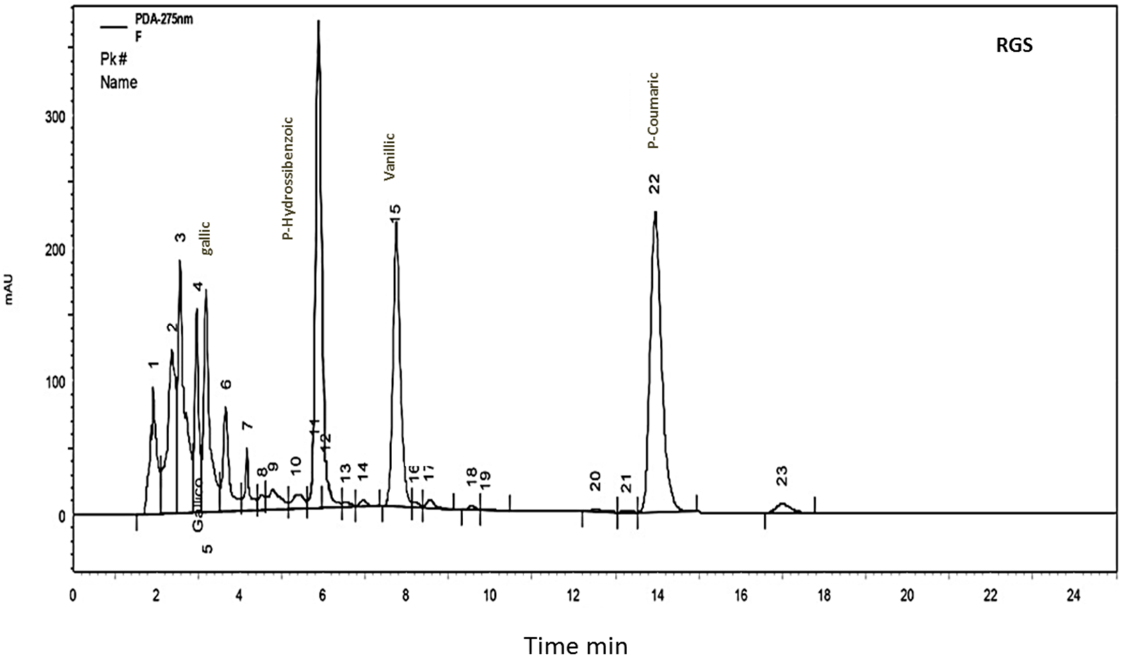

Figure S4. HPLC chromatogram of phenolic acids in Red Grape Skin

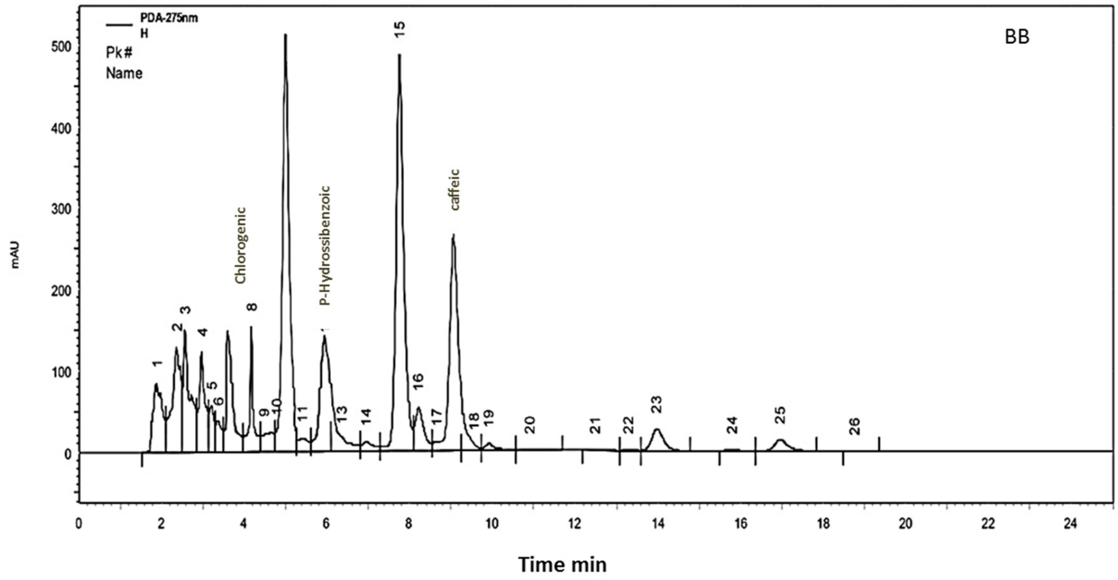

Figure S5. HPLC chromatogram of phenolic acids in Blueberry fruits
